
JOURNAL INFORMATION
==============================
NLM Title Abbreviation: J Clin Lab Anal
Journal ID: J Clin Lab Anal
NLM Journal ID: 8801384
Journal ID: JCLA

Journal of Clinical Laboratory Analysis

ISSN: 0887-8013
EISSN: 1098-2825
Publisher: Wiley

ARTICLE INFORMATION
==============================
PMCID: PMC11474316
DOI: 10.1002/jcla.24975
Article ID: JCLA24975
Article version: 1
Subjects: Issue Information, Issue Information

Issue Information

Electronic publication date: 2023 Oct 9
Publication date: 2023 Aug
Volume: 37
Issue: 15-16
Electronic Location ID: e24975
Copyright: © 2023 The Authors. Journal of Clinical Laboratory Analysis published by Wiley Periodicals LLC.
License: This is an open access article under the terms of the http://creativecommons.org/licenses/by/4.0/ License, which permits use, distribution and reproduction in any medium, provided the original work is properly cited.
License URL: https://creativecommons.org/licenses/by/4.0/

Figure count: 0
Table count: 0
Page count: 2

source-schema-version-number: 2.0
cover-date: August 2023
details-of-publishers-convertor: Converter:WILEY_ML3GV2_TO_JATSPMC version:6.4.9 mode:remove_FC converted:15.10.2024

==============================

Volume 37, Issue 15–16 2023

### JCLA Journal of Clinical Laboratory Analysis

Open Access

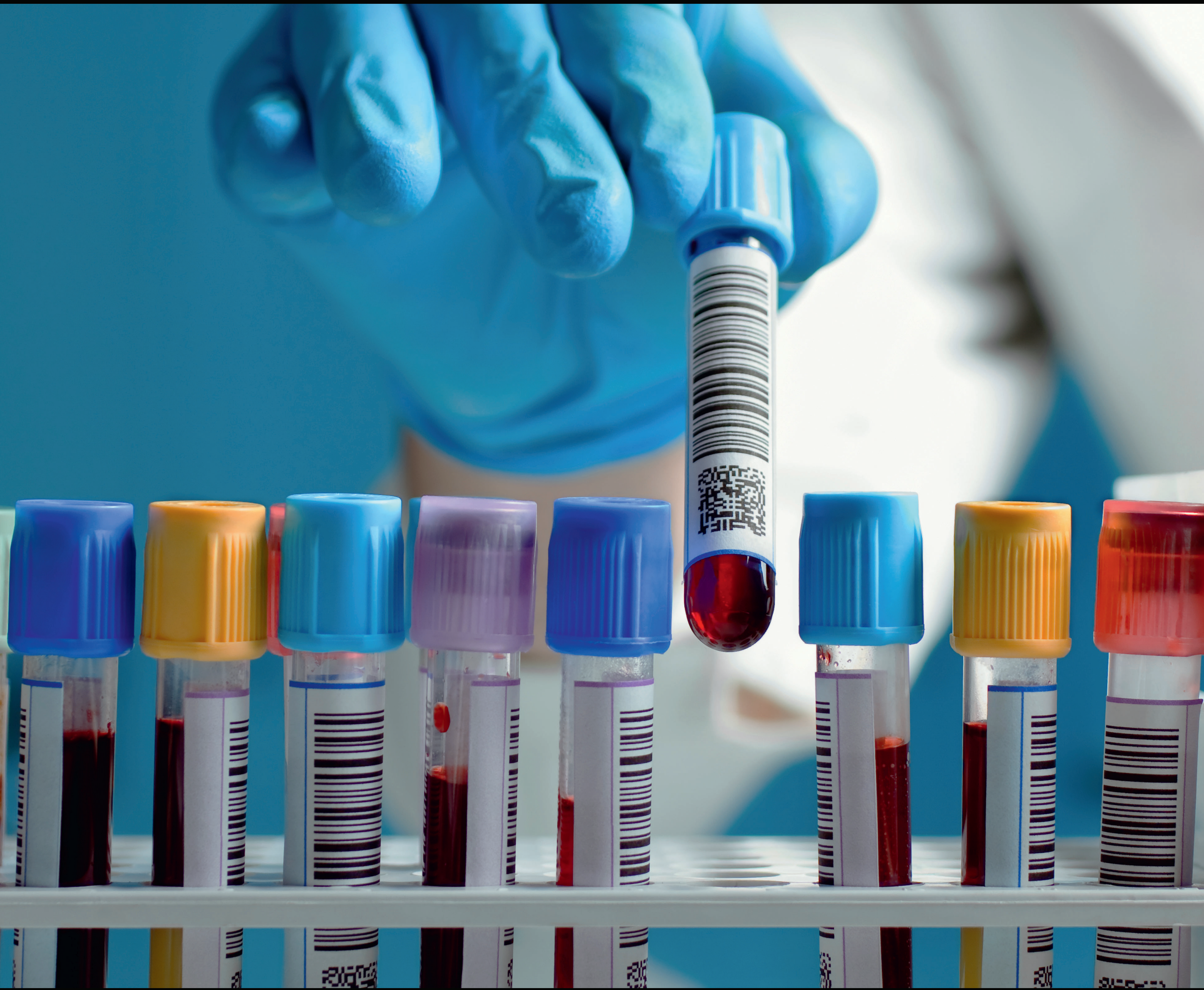

WILEY

### Journal of Clinical Laboratory Analysis

### Incorporating *Diagnostic and Clinical Immunology*

### Editor

**Rong Fu,** General Hospital, Tianjin Medical University, Tianjin, China

### Associate Editors

**Xifeng Dong**
General Hospital of Tianjin Medical University, Tianjin, China

**Chunfang Gao**
Yueyang Hospital of Integrated Traditional Chinese and Western Medicine,
Shanghai University of Traditional Chinese Medicine, Shanghai, China

**Daguang Wang**
The First Hospital of Jilin University, Changchun, China

**Le Yu**
Southern Medical University, Guangzhou, China

**Lin Zhou**
Shanghai Changzheng Hospital, Shanghai, China

### Editorial Board

**Jun Chen**
Tianjin Medical University General Hospital, Tianjin, China

**Amitava Dasgupta**
University of Texas, Texas, US

**Laurence M. Demers**
The Pennsylvania State University, Pennsylvania, US

**Caigan Du**
The University of British Columbia, Vancouver, Canada

**Andrea Ferreira-Gonzalez**
Virginia Commonwealth University, Virginia, US

**Feng Jiang**
Obstetrics and Gynecology Hospital of Fudan University, Shanghai, China

**Rongcai Jiang**
Tianjin Medical University General Hospital, Tianjin, China

**Mark A. Jandreski**
Siemens Healthcare Diagnostics, Inc., New York, US

**Jinhui Liu**
The First Affiliated Hospital of Nanjing Medical University, Nanjing, China

**Ming Liu**
Tianjin Medical University General Hospital, Tianjin, China

**Junxuan (Johnny) Lu**
Penn State College of Medicine, Pennsylvania, US

**Qishui Ou**
Fujian Medical University, Fujian, China

**Roger S. Riley**
Virginia Commonwealth University, Virginia, US

**Jeffrey Schlom**
Center for Cancer Research, National Cancer Institute, Maryland, US

**Xianrang Song**
Shandong Cancer Hospital, Shandong, China

**Gabriel Virella**
Medical University of South Carolina, South Carolina, US

**Wenrong Xu**
Jiangsu University, Jiangsu, China

**Jun-Jun Wang**
The General Hospital of Eastern Theater command, Jiangsu, China

**Xianglin Wu**
Mayo Clinic, Minnesota, US

**Jun Zhang**
Zhejiang University, Zhejiang, China

**Jun Zhou**
Nankai University, Tianjin, China

**Disclaimer:** The Publisher and Editors cannot be held responsible for errors or any consequences arising from the use of information contained in this journal; the views and opinions expressed do not necessarily reflect those of the Publisher and Editors, neither does the publication of advertisements constitute any endorsement by the Publisher and Editors of the products advertised.

Wiley's Corporate Citizenship initiative seeks to address the environmental, social, economic, and ethical challenges faced in our business and which are important to our diverse stakeholder groups. Since launching the initiative, we have focused on sharing our content with those in need, enhancing community philanthropy, reducing our carbon impact, creating global guidelines and best practices for paper use, establishing a vendor code of ethics, and engaging our colleagues and other stakeholders in our efforts.
Follow our progress at www.wiley.com/go/citizenship

**Abstracting and Indexing Services:** The Journal is indexed by Science Citation Index, MEDLINE, and CINAHL. For a complete list of A&I services please visit the journal homepage at www.wileyonlinelibrary.com/journal/jcla.

ISSN 1098-2825 (Online)
View this journal online at www.wileyonlinelibrary.com/journal/jcla

Cover image © angellodeco/Shutterstock
